# A spatial database of colorectal cancer patients and potential nutritional risk factors in an urban area in the Middle East

**DOI:** 10.1186/s13104-020-05310-z

**Published:** 2020-10-02

**Authors:** Neda Firouraghi, Nasser Bagheri, Fatemeh Kiani, Ladan Goshayeshi, Majid Ghayour-Mobarhan, Khalil Kimiafar, Saeid Eslami, Behzad Kiani

**Affiliations:** 1 Department of Medical Informatics, School of Medicine, Mashhad University of Medical Science, Mashhad, Iran; 2 Visualization and Decision Analytics (VIDEA) Lab, Centre for Mental Health Research, Research School of Population Health, College of Health and Medicine, The Australian National University, Canberra, Australia; 3 Department of Gastroenterology and Hepatology, School of Medicine, Mashhad University of Medical Sciences, Mashhad, Iran; 4 Metabolic Syndrome Research Center, Mashhad University of Medical Sciences, Mashhad, Iran; 5 Department of Medical Records and Health Information Technology, School of Paramedical Sciences, Mashhad University of Medical Sciences, Mashhad, Iran

**Keywords:** Colorectal cancer, Geographical information systems, Spatial analysis, Red meat, Dietary fiber, Body mass index

## Abstract

**Objectives:**

Colorectal cancer (CRC) is the third most common cancer across the world that multiple risk factors together contribute to CRC development. There is a limited research report on impact of nutritional risk factors and spatial variation of CRC risk. Geographical information system (GIS) can help researchers and policy makers to link the CRC incidence data with environmental risk factor and further spatial analysis generates new knowledge on spatial variation of CRC risk and explore the potential clusters in the pattern of incidence. This spatial analysis enables policymakers to develop tailored interventions. This study aims to release the datasets, which we have used to conduct a spatial analysis of CRC patients in the city of Mashhad, Iran between 2016 and 2017.

**Data description:**

These data include five data files. The file CRCcases_Mashhad contains the geographical locations of 695 CRC cancer patients diagnosed between March 2016 and March 2017 in the city of Mashhad. The Mashhad_Neighborhoods file is the digital map of neighborhoods division of the city and their population by age groups. Furthermore, these files include contributor risk factors including average of daily red meat consumption, average of daily fiber intake, and average of body mass index for every of 142 neighborhoods of the city.

## Objective

Colorectal cancer (CRC) is the third most frequently diagnosed malignancy and the second most common cause of death from cancer worldwide [1, 2]. CRC incidence varies in the world with the highest incidence rates in Australia, New Zealand, Europe, and North America and the lowest in Africa and South-Central Asia [1, 3]. The incidence rate of CRC was 7–8 per 100,000 for both males and females in Iran from 1996 to 2000 [4]. However, this incidence rate has been increased to 11.8 and 16.5 (per 100,000) for females and males in 2014 [5]. This increasing trend in CRC incidence may related to high rate of urbanization, people’s lifestyle and diet change [5, 6].

Both environmental and lifestyle factors contribute to the risk of CRC. Some important such factors include age, high body mass index (BMI), high-fat diet, alcohol consumption, smoking, consumption of red meat, low intake of vegetables and fruit (fiber intake) [2, 7]. Spatial analysis of CRC incidence may provide a new knowledge on the relationships between environmental risk factors and people lifestyle with CRC burden across communities. This will enable policymakers to develop tailored intervention to areas where the CRC risk is greater. Thus, we investigated the spatial variation of CRC incidence in the city of Mashhad Iran [8]. In that study, we used Local Moran’s *I* statistic (an spatial local clustering approach) [9] to identify high-risk and low-risk areas. A linear regression model developed to quantify the relationship of CRC occurrence with common risk factors [10] including age [2, 11], BMI [12–14], daily red meat consumption [15–20] and daily fiber consumption [7, 20–22]. We developed a comprehensive spatial dataset linked to other attribute data and we would like to offer this dataset for further investigation in future spatial analysis of CRC incidence in Mashhad and elsewhere.

## Data description

Geographic Information System (GIS) is a powerful tool for visualizing spatial variation and cluster detection in the pattern of CRC incidence to identify unmet areas [23]. GIS can link geo-referenced risk factors and CRC incidence data with other spatial and temporal data to investigate spatial clustering across time and space [24]. Data were extracted from three different databases. Individual CRC cases were obtained from the population-based cancer registry in Khorasan-Razavi Province. There were 695 CRC diagnosed cases in the city of Mashhad between March 2016 and March 2017. This data set contains patients addresses in the Persian language which had to be geocoded manually using the software Google MyMaps (https://www.google.com/mymaps). These geo-coded data were subsequently transformed into a Keyhole Markup Language (KML) file and imported to ArcGIS software version 10.6 (ESRI, Redands, CA, USA) for further spatial analysis. We randomly jittered the latitude and longitude of the patients address into a 100-m buffer to avoid potential identification of CRC cases. The neighborhood divisions and their population separated in age groups were provided from the City Council in Mashhad. The age groups were presented in the categories including, 0–4, 5–9, 10–14, 15–19, 20–24, 25–29, 30–34, 35–39, 40–44, 45–49, 50–54, 55–59, 60–64, and over 65. The age data were provided for both gender (male and female separately). Data regarding risk factors like BMI and average of daily consumption of red meat and fibers, were obtained from the MASHHAD cohort study [25], between 2010 and 2020. The original CRC cases data were visualised as point data in Mashhad. We used spatial interpolation technique and calculate the data for each suburb of the city.

Anselin Local Moran’s *I* statistic was used to identify the potential clusters in CRC pattern at the neighborhood level based on incidence rate. The CRC incidence rate was calculated by total population and the frequency of cases per 100,000 persons in each neighborhood in Mashhad. This method helps to find high–high (regions as similar clusters with high values) and low–low (regions as similar clusters with low values of CRC incidence), and high–low (HL) and low–high (LH) areas as special outliers with dissimilarity. We used linear regression model to analyse the relationship between CRC incidence and the risk factors of CRC. In this method, we considered CRC frequency as the dependent variable, and the proportion of the population over 50 years of age, average BMI, average consumption of daily red meat, and average of daily fiber intake as independent variables. The coefficient of determination (R^2^) was used to establish the performance of regression model [8]. Researchers can link other environmental risk factors such as air pollution and heavy metals to this dataset and investigate their impact on CRC incidence. Table 1 shows the details of each dataset and provides links to access them.

**Table 1:** Overview of data sets

Label	Name of data file/data set	File types (file extension)	Data repository and identifier (DOI or accession number)
Data file 1	CRCcases_Mashhad	Shape file (.shp)	Harvard Dataverse (https://doi.org/10.7910/DVN/RFOCK7) [26]
Data file 2	Mashhad_Neighbourhoods	Shape file (.shp)	Harvard Dataverse (https://doi.org/10.7910/DVN/RFOCK7) [26]
Data file 3	Avg_Daily_Red_Meat_Consumption_Mashhad	Shape file (.shp)	Harvard Dataverse (https://doi.org/10.7910/DVN/RFOCK7) [26]
Data file 4	Avg_Daily_Fiber_Consumption_Mashhad	Shape file (.shp)	Harvard Dataverse (https://doi.org/10.7910/DVN/RFOCK7) [26]
Data file 5	Avg_BMI_Mashhad	Shape file (.shp)	Harvard Dataverse (https://doi.org/10.7910/DVN/RFOCK7) [26]

## Limitations

The coverage and precision of population-based cancer registry in Iran are not 100% accurate due to insufficient electronic registries, so we may have missed some CRC patients in our study. However, the detection of high-risk and low-risk areas should not be affected by this limitation.

## Data Availability

The data described in this data note can be freely and openly accessed on the Harvard Dataverse under (https://doi.org/10.7910/DVN/RFOCK7) [26]. Please see Table 1 and reference list for details and link to the data.

## Abbreviations

CRC: Colorectal cancer
ASR: Age standardized rate
BMI: Body mass index
OLS: Ordinary least squares
GIS: Geographic Information System
KML: Keyhole Markup Language
HH: High–high
LL: Low–low
HL: High–low
LH: Low–high
MSH_NBH: Mashhad neighborhoods
All_0_4: Population between 0 and 4 for both genders
M_0_4: Population between 0 and 4 for males
F_0_4: Population between 0 and 4 for females
Avg_DRMC: Average of daily red meat consumption (g)
Avg_DFC: Average of daily fiber consumption (g)
Avg_BMI: Avearge of body mass index (kg/m^2^)
